# Sample-Guided Adaptive Class Prototype for Visual Domain Adaptation

**DOI:** 10.3390/s20247036

**Published:** 2020-12-09

**Authors:** Chao Han, Xiaoyang Li, Zhen Yang, Deyun Zhou, Yiyang Zhao, Weiren Kong

**Affiliations:** School of Electronics and Information, Northwestern Polytechnical University, Xi’an 710129, China; hanc@mail.nwpu.edu.cn (C.H.); nwpuyz@mail.nwpu.edu.cn (Z.Y.); dyzhou@nwpu.edu.cn (D.Z.); zhaoyiyang@mail.nwpu.edu.cn (Y.Z.); k@mail.nwpu.edu.cn (W.K.)

**Keywords:** domain adaptation, adaptive class prototype, sample selection

## Abstract

Domain adaptation aims to handle the distribution mismatch of training and testing data, which achieves dramatic progress in multi-sensor systems. Previous methods align the cross-domain distributions by some statistics, such as the means and variances. Despite their appeal, such methods often fail to model the discriminative structures existing within testing samples. In this paper, we present a sample-guided adaptive class prototype method, which consists of the no distribution matching strategy. Specifically, two adaptive measures are proposed. Firstly, the modified nearest class prototype is raised, which allows more diversity within same class, while keeping most of the class wise discrimination information. Secondly, we put forward an easy-to-hard testing scheme by taking into account the different difficulties in recognizing target samples. Easy samples are classified and selected to assist the prediction of hard samples. Extensive experiments verify the effectiveness of the proposed method.

## 1. Introduction

**Figure 1:**
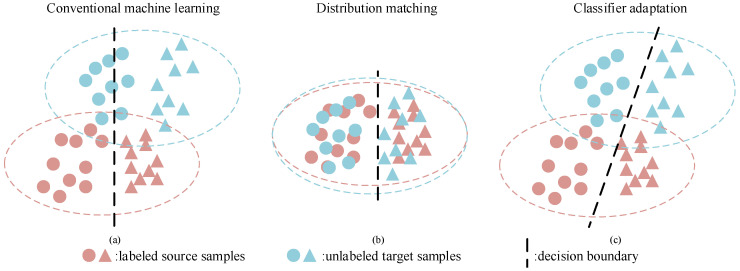
Different measures for cross-domain recognition tasks.

A fundamental assumption of conventional machine learning is that test samples are drawn from the identical distribution with training samples [1]. However, such an assumption does not always hold in real-world applications. For multi-sensor systems, the issue of distribution mismatch can be caused by many factors, e.g., various sensor parameters and background noises. Such a mismatch can be easily found in remote sensing images (different area and weather condition) and person re-identification (different shoot angle). The violation of the assumption results in severe performance degradation, and labeling data from all sources is laborious; as Figure 1a shows, the decision boundary induced by source samples performs poorly in the target domain. To tackle this problem, domain adaptation (DA) [2] has attracted much attention. DA aims at leveraging rich knowledge from training data (also referred to as the source domain) and making decisions about different, but related testing data (also referred to as the target domain), which has been successfully applied in many areas, such as image classification [3,4,5], person re-identification [6,7], and activity recognition [8,9,10].

In order to address the issue of distribution mismatch, a series of research works focused on discovering domain-shared feature representations [11,12], then we can train a classifier with the learned representation. A graphical illustration of the idea is proposed in Figure 1b; intuitively, the decision boundary learned with source samples also works well in the target domain since they are aligned. Instance re-weighting aims to narrow the distribution distance by assigning weights to samples, the emphasis being different criteria for calculating the weights, e.g., kernel mean matching (KMM). Chen et al. re-weighted source samples for subspace alignment, and the weight $w_{i}$ of source sample $x_{i}$ increases if it has a similar distribution as the target samples [13]. Chu et al. attempted to match distributions with KMM and minimize the empirical risk of source domain simultaneously [14]. However, studies show that it works well in the scenario where the source and target domains have few differences, and the performance degrades as the domain gap becomes larger [15]. Feature mapping seeks the appropriate feature space/subspace to reduce domain discrepancy. Compared to simply re-weighting, it is capable of learning more powerful representations by means of complex non-linear mappings, such as kernel methods and deep neural networks, thus yielding remarkable performance. Pan et al. proposed a PCA-like framework, named transfer component analysis (TCA), which adopts the maximum mean discrepancy (MMD) as the loss function and maps data to the feature space [16]. Extending TCA, Long et al. introduced conditional MMD by computing the pseudo labels of target samples, and an iterative training scheme was applied to obtain more accurate labels [17]. Inspired by the success of deep neural networks (DNN), the combination of domain adaptation and DNN also achieves dramatic success. Tzeng et al. proposed a composite network that minimizes the distribution distance by MMD and the cross-entropy of source samples [18]. Furthermore, Long et al. employed multi-kernel MMD for matching features [19]. Both instance re-weighting and feature mapping rely greatly on the evaluation of the distribution distance; however, statistical metrics, e.g., MMD, have been proven to be sensitive to outliers and class weight bias [20]. Another popular method is adversarial training based domain adaptation, which constructs a source classifier and a domain discriminator simultaneously. The source classifier aims to recognize the objects of multiple classes, and the domain discriminator is designed to learn consistent features for two domains.

Despite the success of distribution matching, there still remains two major issues: (1) Is it necessary for distribution matching? Deep convolution neural networks (CNN) can learn fairly unbiased representations for image data, which is shown by the fact that they report high accuracy over complex vision tasks using CNN representations and a linear classifier [21]. (2) Existing works made predictions for target samples independently, i.e., the label $y_{i}$ of target sample $x_{i}$ is obtained according to the relation between $x_{i}$ and source samples $X_{s}$, while ignoring the relations within target samples. In this paper, we propose a novel framework based on the nearest class prototype for unsupervised domain adaptation. Similar to instance re-weighting, the proposed method aims to study the sample difference (both in the source and target domain, while instance re-weighting focuses on source samples) in cross-domain scenarios. As Figure 1c shows, instead of aligning domains, we aim at learning the adaptive decision boundary for two domains. Specifically, we explore the diversity within samples belonging to the same class and find a balance between single-sample discrimination and class wise discrimination. Furthermore, a multi-stage training scheme is presented for better exploiting the discriminative structures in the target domain. The contributions of this paper are summarized as follows.

- Corresponding to Issue (1), there is no distribution matching strategy in our method. Experimental results show that the proposed classifier adaptation can achieve comparable performance when compared to popular distribution matching methods.
- In response to Issue (2), we propose an easy-to-hard testing scheme. The underlying idea is that the difficulties in recognizing target samples vary from each other, and easy samples along with their labels can assist the prediction for hard samples.
- We propose the modified nearest class prototype, which allows more diversity within the same class. Ideally, clusters with less domain discrepancy would yield correct predictions for target samples.

The rest of this paper is organized as follows. Section 2 gives the background knowledge on related DA works. Then, the nearest neighbors and nearest class prototype are discussed. In Section 3, we describe our method in detail. Section 4 and Section 5 present the experiments and some empirical analysis. Finally, Section 6 concludes the paper and provides some ideas for future research.

## 2. Related Works

In this section, we first give a formal definition for unsupervised/homogeneous domain adaptation. Then, a brief introduction of the nearest neighbors and nearest class prototype is presented.

### 2.1. Domain Adaptation

Domain adaptation deals with the scenario where training and testing data have different distributions. Formally, we first introduce the concepts of domain and task.

Domain: A domain consists of data and a distribution, D={X,p(X)}. For standard DA problems, we have $D_{s}$ and $D_{t}$ for the source and target domain, respectively. Besides, two domains have different distributions, $p\left(X_{s}\right)\neq p\left(X_{t}\right)$.

Task: A task includes labels and the mapping function, T={Y,f(·)}. It is worth noting that we can learn multiple source mappings $f_{s}$ with different models since source data are well labeled. Correspondingly, we have $T_{s}$ and $T_{t}$, and the goal is to learn the target mapping $f_{t}\left(\cdot \right)$, i.e., $f_{t}\left(X_{t}\right)=Y_{t}$.

In this paper, we study unsupervised/homogeneous domain adaptation problems, which means that (1) there is no labeled samples for training in the target domain, and target labels are only available for evaluating methods $Y_{t}=\emptyset$. (2) Source and target data have the same dimensions $X_{s},X_{t}\in R^{m}$. Previous works focused on reducing the distribution mismatch, based on either nonparametric (MMD, CORAL) or parametric (A-distance) metrics, but gave very limited considerations on the relation between learned representation and the decision boundary. A similar idea to ours is pseudo-label based domain adaptation (PLDA), which alternates between feature learning and pseudo-label learning. To our best knowledge, Joint Distribution Adaption (JDA) [17] is the first method that adopts a classifier to obtain pseudo labels, and the objective is to estimate the conditional probability of target samples. Consequently, it allows us to match both the conditional and marginal distribution. Besides, it has an iterative updating strategy, and the classifier is expected to be more powerful as the training goes. Wang et al. further pointed out that pseudo-labels are not always reliable because of the domain shift, then proposed confidence-aware pseudo label selection (CAPLS) [22] and selective pseudo labeling (SPL) [23], which select more credible pseudo labels.

Such methods combine feature learning and classifier training, and the desired representations and classifiers can be obtained simultaneously. In this paper, we aim to learn a domain adaptive classifier based on the nearest class prototype, which differs from PLDA in the following aspects: (1) PLDA does not consider the characteristics of a classifier; in other words, it does not care about which type of classifier one chooses: the nearest neighbor is fine, and support vector machine is also feasible. The classifier is only used to make predictions; the emphasis is to learn domain-invariant representations. However, for our method, we analyze how the domain discrepancy would affect the standard nearest class prototype and propose several strategies to alleviate the negative effects. Most importantly, there is no distribution matching procedures in our method, which makes it differ most compared to other works. (2) It seems that our easy-to-hard testing scheme is similar to sample selection, since they all have a selection process. In fact, they are completely different. Selective pseudo labeling aims to reduce the negative impacts of the wrong labels, and our goal is to better exploit the discrimination power, i.e., we can obtain more precise predictions for hard samples by considering both source (all) and target (easy) samples. Hence, sample selection needs to predict all the target samples during each iteration, but our easy-to-hard testing adopts a decreasing number of target samples: once a sample is deemed to have a credible label, it will be removed from the test set.

### 2.2. Nearest Neighbor and Nearest Class Prototype

The nearest neighbor (NN) and nearest class prototype (NCP) are two pattern recognition methods, which are widely used due to their simplicity [24,25,26]. Given a query sample $x_{q}$ and a training set [X,y(X)], under the clustering assumption and manifold assumption, NN and NCP search for the nearest sample/class prototype and take the corresponding label as the prediction for the query sample. It is worth emphasizing that there are many ways to compute the class prototype [27], e.g., learning vector quantization (LVQ) [28] and mean vector (MV). In this paper, we focus on MV due to its simplicity and stationarity.

**Figure 2:**
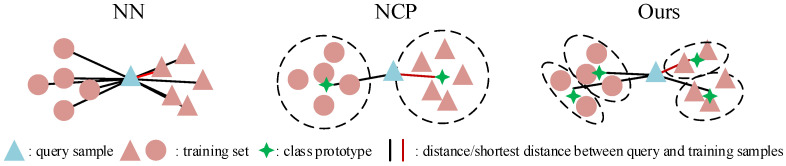
A graphical illustration of the nearest neighbor, nearest class prototype (NCP), and the proposed modified NCP. NN searches for the nearest samples, and NCP seeks the nearest class prototype, while our modified NCP finds a balance between them. Samples from each class are first clustered into several groups, and the goal is to find the nearest group center.

In Figure 2, we give an intuitive description of the NN and NCP. These methods work well when the training and testing set have the same distribution, but when applied in cross-domain tasks, the clustering assumption does not always hold due to distribution mismatch, thus leading to severe performance degradation. In this paper, we follow the assumption of sample re-weighting based methods, i.e., samples have different importance. A modified NCP method is proposed, which allows more diversity within one class than the original NCP, and a detailed description can be found in the next section.

## 3. Methodology

In this section, we first introduce the general framework of the proposed method and the necessary notations, then two components, the modified NCP and easy-to-hard testing scheme, are described in detail.

### 3.1. Framework and Notations

Suppose that we have source and target data $X_{s}/X_{t}\in R^{n_{s}/n_{t}\times m}$, which are drawn from two related, but different distributions $p\left(X_{s}\right)/p\left(X_{t}\right)$. Besides, source data are well labeled ($Y_{s}\in R^{n_{s}\times 1}$ is available), while target data have no labels ($Y_{t}\in R^{n_{t}\times 1}$ is not available). The goal is to obtain labels for target samples with high precision. Table 1 introduces the necessary notations and descriptions.

**Figure 3:**
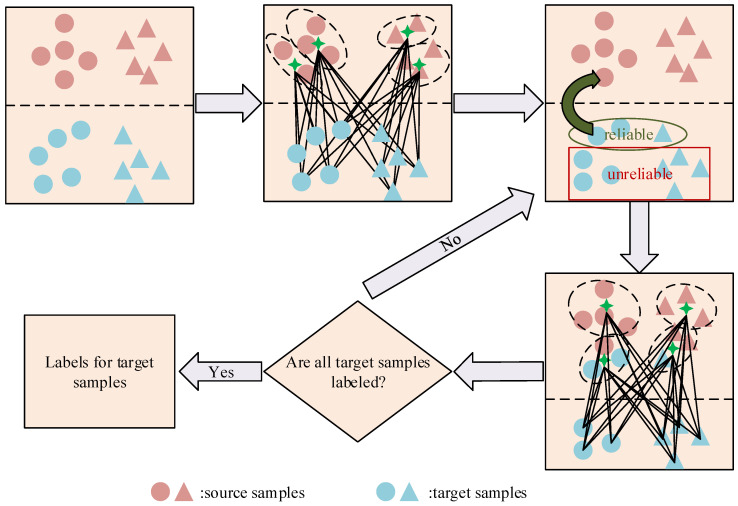
A graphical illustration of the total framework. First, we use the labeled source samples to construct a basic classifier, then the easy samples from the target domain are selected by considering the confidence. Adding easy samples along with their labels to the training set, a more powerful classifier can be obtained. The process continues until every target samples has been labeled.

In this paper, we propose a novel unsupervised DA solution based on the NCP. Firstly, we present the modified NCP by finding a balance between single-sample discrimination and class wise discrimination. It allows more diversity within the same class, thus, it is capable of preserving more local structures. Besides, an easy-to-hard testing scheme is introduced. Instead of predicting target samples independently, it selects so-called easy samples, which are considered to have more confidence about the predicted labels, and easy samples along with their labels can assist the prediction of hard samples. By doing this, we hope to utilize the discriminative information existing in target samples. A graphical illustration of the proposed method is given in Figure 3.

As the title states, we aim to explore the intrinsic relation among samples and learn more robust decision boundaries, both in the source and target domain. To be more specific, we consider the domain discrepancy revealed in each source sample. Recall that NCP exploits the class center to make the prediction for query samples, but for each class, there are both less biased and more biased samples. Intuitively, we hope to use the less biased samples for prediction. By clustering source samples drawn from the same class into several clusters (class sub-center), the query samples are capable of selecting the closest class sub-centers, thus alleviating the negative effects brought by very biased samples. When it comes to the target domain, we hope to make precise predictions by utilizing local discriminative structures existing in the target domain. Previous works focused on the discriminative information in the source domain, which is easy to quantize and use since source samples are well labeled, while ignoring the discrimination power among unlabeled target samples. Similar to the source, we assume target samples have different difficulties in their prediction. Then, a hierarchical structure is presented naturally, and easy samples are predicted in the earlier steps, so we can label hard samples by considering both labeled source samples and previously predicted easy target samples.

### 3.2. Modified Nearest Class Prototype

The nearest neighbor selects the nearest samples to a query sample by means of certain metrics, e.g., the Euclidean distance. It explores the sample-to-sample relation, so we call it single-sample discrimination, which can be considered as a specific form of the manifold assumption. However, when applied in DA tasks, the manifold assumption may not always hold due to the domain shift [29], thus causing performance degradation. On the other hand, the nearest class prototype follows the clustering assumption and makes the prediction by means of the distance between the class center and the query sample, so we call it class-wise discrimination. Similarly, the clustering assumption is also broken by the domain shift [29]. In this paper, we propose the modified nearest class prototype. The original NCP considers all the training samples to be equal, while ignoring the influence brought by the distribution match, i.e., samples have different importance since they hold various degrees of bias. Naturally, we hope to predict the target sample by less biased source samples.

We can quantify the bias degree of two sets of data, i.e., the source set and target set, using some parametric or nonparametric metrics. However, evaluating the degree of bias for certain sample is not feasible. Therefore, we further assume that the less biased and very biased samples exhibit disparate properties, then they can be represented by several clusters. It is worth emphasizing that the distribution mismatch would ruin the cross-domain manifold assumption and clustering assumption, but when it comes to within-domain cases, the manifold assumption and clustering assumption always hold since all samples drawn from the same domain are considered to be independent and identically distributed (i.i.d.).

Given training data and labels [$X_{train},Y_{train}$] and testing data $X_{test}$, the pseudocode is shown in Algorithm 1. *k* is the number of clusters for a certain category. Firstly, for samples with the same labels in the training set, we divide them into *k* centers, then record each cluster $cluster_{i}\in R^{k\times m}$. Here, we employ K-means clustering due to its simplicity. When testing, we calculate the distance among the query sample and each cluster (there should be c·k clusters in total). Then, the label can be obtained by the cluster with the shortest distance. Notice that we set a fixed value *k* as the number of clusters for each class, which seems unreasonable. The number should be changed as the distribution changes. If the samples are close to each other, we need a small *k* to keep their affinity, and vice versa. We admit that it is not a good choice for a fixed *k*, but it also shows the following advantages. (1) There is no need for a search program for *k*, thus keeping it efficient. According to the literature, searching for an optimal *k* is somewhat time-consuming, e.g., the elbow method and average silhouette width demand multiple operations of K-means [30]. Even worse, there may be no such thing as optimal *k* [31]. (2) We witnessed enough performance improvements with it, so our method achieves state-of-the-art performance when compared with existing works. Finally, we compute the confidence score $C_{test}$ for each target sample by normalizing the distance, which can be used as the criterion of sample selection.
**Algorithm 1:** mNCP: modified nearest class prototype.
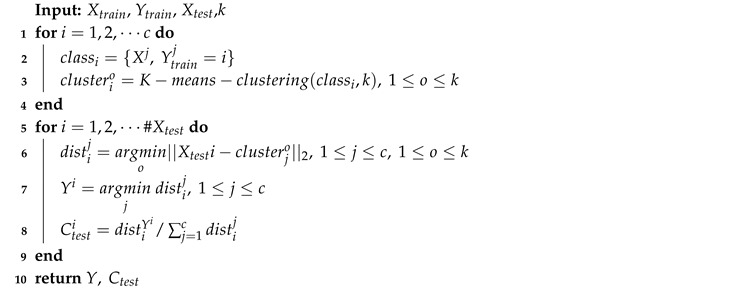


### 3.3. Easy-To-Hard Testing Scheme

Existing works make predictions independently in the target domain, which only investigates the cross-domain correlation, i.e., for a certain target sample *x*, the prediction is obtained based on the relation among *x* and source samples $X_{s}$, but the relation among *x* and other target samples $X_{t}$ is ignored. Recall that the manifold and clustering assumption still hold within-domain, so naturally, the idea of preserving local structures in the target domain is raised. In this paper, we propose an easy-to-hard testing scheme; specifically, it has a hierarchical prediction strategy. Samples in the target domain are considered to have different difficulties in their prediction, then we select the so-called easy samples (which are believed to have more confidence) in the early stages during training, and these samples along with their more confident labels are taken into consideration for predicting hard samples. The underlying philosophy is that we can obtain precise labels for easy samples based on the cross-domain relation, and for hard samples, we need to combine the cross-domain and within-domain relations to predict them. It is worth emphasizing that determining which sample is easy (or hard) is difficult to some extent. Here we first construct a basic classifier with labeled source samples, then we can select samples with a much confidence as easy samples.

The detailed calculation can be found in Algorithm 2. Here, we split the concept of the target/source domain and the training/testing set. For initialization, the source data are set to be the training set, and the target is for the testing. Then in each iteration, we select *N* samples with the highest confidence. It is worth noting that there are two ways to determine *N*, i.e., hard threshold or fixed number. We chose the fixed number, because selecting a proper threshold is laborious. Thinking of the worst case, if we employ a relatively big value, the model would stop. However, if we utilize a fixed number (>0), the model can always converge. To be more specific, we set a hyper-parameter iter as the number of total iterations, then *N* can be determined by $N=n_{t}/iter$. Finally, the selected samples $X_{test}^{index}$ along with their labels $Y^{index}$ are integrated into the training set $X_{train}/Y_{train}$. It is worth noting that once a sample is selected as an easy sample, its label is accepted, which means that we do not need to predict it again. Therefore, the size of the testing set decreases, and that of the training set increases (corresponding to Algorithm 2 Lines 6–8). After several iterations, all target samples are considered to be well labeled.
**Algorithm 2:** Easy-to-hard testing.
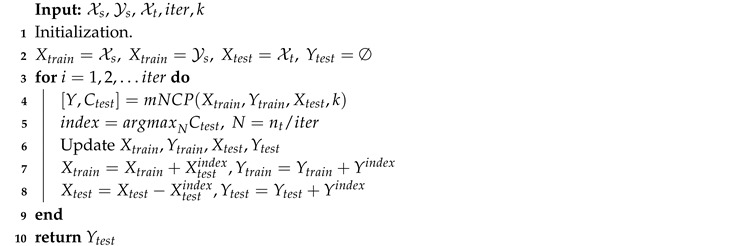


## 4. Experiments

In this section, we give the detailed description of the experiments. Firstly, we introduce two widely-used datasets for DA problems, i.e., Office-Caltech10 and ImageCLEF (Cross Language Evaluation Forum (CLEF)), followed by competing methods and the parameter setting. Then, the numerical results are presented with some analysis. Besides, we conduct experiments for parameter sensitivity.

### 4.1. Data Preparation

ImageCLEF (https://www.imageclef.org/2014/adaptation) is a visual competition, held by the Cross Language Evaluation Forum (CLEF). It consists of three domains, Caltech (C) [32], ImageNet (I) [33], and Pascal (P) [34]. There are twelve classes of objects for each domain, e.g., airplane, bike, bird, boat, bottle, bus, car, dog, horse, monitor, motorbike, and people. Besides, the number of images per category is 50.

Office-Caltech10 [35] includes four domains, Amazon (A), Caltech (C) [32], webcam (W), and DSLR (D). There are ten classes of objects and 8-151 images per class. The accurate number of images for each category can be found in Table 2.

**Figure 4:**
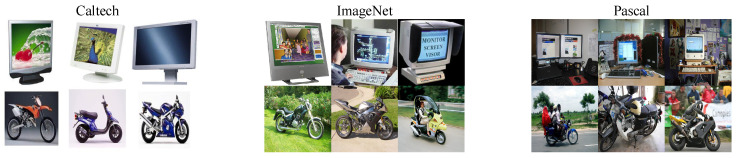
Cross-domain recognition.

Given two random domains, e.g., Amazon (A) and webcam (W), we can construct two DA tasks, A→W and W→A. Consequently, we can construct $A_{3}^{2}=6$ tasks for ImageCLEF and $A_{4}^{2}=12$ tasks for Office-Caltech10. A graphical description of the cross-domain tasks is shown in Figure 4.

### 4.2. Experimental Setting

We compare the proposed method with two baseline models and four state-of-the-art DA methods, which are listed below.

- Nearest neighbor (NN): NN is selected as a baseline for examining the effectiveness of the proposed method.
- Nearest class prototype (NCP): Similar to NN, NCP is also a baseline method since the proposed method is highly correlated with them.
- Confidence-aware pseudo label selection (CAPLS): CAPLS (proposed in IJCNN2019 [22]) selects reliable labels by confidence and learns transferable representations across domains.
- Modified A-distance sparse filtering (MASF): MASF (proposed in Pattern Recognit.2020 [36]) presents an l2 constraint as the metric of domain discrepancy.
- Generalized soft-max (GSMAX): GSMAX (proposed in Inf.Sci.2020 [37]) aims at learning smooth representations and decision boundaries simultaneously.
- Selective pseudo labeling (SPL): SPL (proposed in AAAI2020 [23]) is also a selective pseudo labeling strategy based on structured prediction.
- Discriminative sparse filtering (DSF): DSF (proposed in Sensors 2020 [38]) combines discriminative feature learning and distribution matching based on sparse filtering.

For CAPLS, we set the number of iterations T=10 and the feature dimension k=100. For MASF, we set the balance factor α=0.001 and the feature dimension k=100. For GSMAX, we set the balance factor β=0.05,γ=0.0001, and the number of nodes α=8 on ImageCLEF and α=10 on Office-Caltech10. For SPL, we set the number of iterations T=11 and the feature dimension k=100. For our method, we set the number of source clusters k=5 and the number of iterations iter=5. Besides, for all methods, we adopted the Resnet50 features for ImageCLEF and Decaf6 features for Office-Caltech10. The deep models were pre-trained on ImageNet without fine-tuning, and no pre-processing strategy was applied.

Following the setting of [23,38], we report the classification accuracy on the target data as the evaluation metric.

(1) $$\begin{array}{cc} Accuracy & =\frac{\sum_{i=1}^{n_{t}}1\left(\hat{y}\left(x_{i}\right)=y\left(x_{i}\right)\right)}{n_{t}},\;x\in X_{t}\\ 1(case) & =\left\{\begin{array}{cc} 1 & ,\;case\;is\;TRUE\\ 0 & ,\;otherwise \end{array}\right. \end{array}$$

where $\hat{y}$ denotes the predicted label and *y* is the true label, so 0%≤Accuracy≤100%.

### 4.3. Implementation Details

For the reproducibility of the paper, here we report the experimental details.

(1) All datasets (original images and extracted features) and part of the code (CAPLS, SPL) can be found in public GitHub repositories, and the link is shown in the Acknowledgments.
(2) We chose the no pre-processing strategy for the extracted features; as mentioned earlier, they are good enough for recognizing.
(3) All methods were implemented with MATLAB 2017a. To eliminate the effect of random numbers, we fixed the random seed to zero.
(4) We are pleased to share our code if anyone is interested; please contact Chao Han (hanc@mail.nwpu.edu.cn).

### 4.4. Results

We report the numerical results in Table 3, where the boldface denotes the highest accuracy. To sum up, the proposed method achieves state-of-the-art performance with respect to the average accuracy. Furthermore, we have the following observations:

- OURS vs. NN, NCP: Compared to these two baseline models, our method is significantly better. NN and NCP have no adaption measures; thus, they would be heavily affected by distribution mismatch. According to the results, our method yields better recognition accuracies on almost every sub-task, and the improvements could be higher than 15% on some tasks, e.g., D→A and D→C. This findings confirm that the proposed modified NCP and easy-to-hard testing can help make more robust predictions on cross-domain tasks.
- OURS vs. MASF: OURS is superior to MASF. MASF proposes the modified A-distance for marginal distribution matching; however, it has limited considerations on the relation between the learned representations and the decision boundary. On the contrary, our method tries to adjust the decision boundary adaptively. Consequently, our method achieves superior performance.
- OURS vs. CAPLS, SPL: These two methods assign pseudo labels on target samples and select highly confident ones, then an iterative feature aligning strategy is applied to learn the transferable representations. Pseudo labels for target samples allow them to match the conditional probability distribution across domains, so that the learned representations are more discriminative than MASF. However, they still fail to explicitly model the relation between features and classifiers. Besides, they are easily influenced by the quality of pseudo labels. From the results, we can see that MASF < CAPLS, SPL < OURS (with respect to average accuracy).
- OURS vs. GSMAX: Objectively speaking, our method works better than GSMAX, which can be considered to be the closest method to ours. It learns a dynamic decision boundary by thinking about both labeled source samples and unlabeled target samples, the underlying idea of which is all samples (including source and target samples) should be far away from the decision boundary. Compared to our method, it does not give consideration to the difficulties of target samples and integrates them all into training; naturally, the wrongly-labeled sample would have negative effects for final recognition.
- OURS vs. DSF: DSF performs slightly worse than the proposed method. DSF explores feature separability and distribution matching simultaneously, while it only adopts a linear regression-like constraint for computing efficiently. Such a constraint cannot handle the complex feature distribution, especially for high-dimensional features. Our method aims to find the optimal classifier, rather than feature transformation, thus obtaining higher accuracies. Besides, we also report the running time of these methods; our method also runs faster than it.

It is somewhat surprising that the baseline methods (NN, NCP) achieve comparable or even superior performance when compared to state-of-the-art DA works on several subtasks, e.g., I→P. Does this mean that the study of domain adaptation is meaningless? We think the answer is absolutely no. Firstly, strong evidence of the improvements brought by DA works can be found when referring to average accuracy, which reveals the greater robustness of DA works from a statistical point of view. Secondly, the theorem of no free lunch [39,40] indicates that there is no algorithm that can be chosen as the best choice for all problems. Naturally, it is reasonable that baseline methods perform better that state-of-the-art ones (for a few cases).

Recall that instance based methods would be heavily affected by increasing gaps; however, the results show that the proposed method could alleviate the negative effects to some extent. Here, we use the accuracy obtained by non-adaptation methods to measure the domain gap. These methods are expected to have better results in the scenario where testing and training data have the same distribution. Since the proposed method is a variant of the nearest class prototype (NCP), we use the NCP as the non-adaptation method. If the NCP achieves high accuracy on a task, this means that there are few domain gap for this task, and vice versa. We can say that task C→W has a larger domain gap than task C→D since the NCP achieves higher accuracy on the task C→D (82.17% vs. 76.95%). However, the proposed method has larger improvements on C→W (76.95%→89.83%, 13%↑) than C→D (82.17%→87.26%, 5%↑).

Another thing to notice is that when we have very few training samples, i.e., D→C, D→W, and D→A, constructing too many clusters would degrade the proposed method to the nearest neighbors. For example, domain DSLR has only eight images of mug, but we still build five clusters for this class, then each cluster holds only 1 4 images. Therefore, the performance of mNCP is expected to be close to NN. However, according to Table 3, our method still gains more than 15% improvements on average accuracy for D→C and D→A, and these results further support the idea of easy-to-hard testing. Although we may not have enough samples in the earlier stages, as the hierarchical testing goes, more samples are integrated into the training set, then we are capable of exploring local discriminative structures.

### 4.5. Parameter Sensitivity Analysis

For better understanding the proposed method, we investigate how each hyper-parameter affects the performance by setting them to a series of different values while fixing the other one. Our method has two hyper-parameters, the number of source clusters *k* and the number of iterations iter.

**Figure 5:**
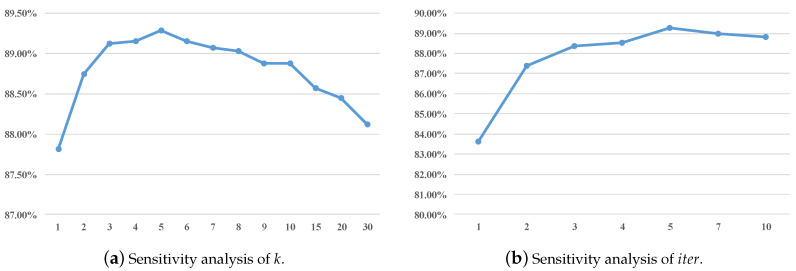
Sensitivity analysis. X-axis: the value of the parameters. Y-axis: the mean accuracy (%) on two datasets.

Sensitivity analysis of *k*: *k* indicates how many clusters we need for training. When k=1, this means that we think all the training samples are equally important, and the method degrades to NCP. As *k* becomes larger, more diversity is allowed within the same class. Our method is able to explore the different importance for training samples. However, if it is too large, especially for the extreme case, k=#train, the method degrades to NN, which is sensitive to outliers and easily affected by distribution mismatch. As shown in Figure 5a, the mean accuracy first arises then falls, which is consistent with our analysis.

Sensitivity analysis of iter: iter is the total iterations for target data. When iter=1, our method do not adopt the easy-to-hard testing scheme. We can see that the mean accuracy increase 4% immediately as setting iter to 2, this finding verifies that the discriminative structure within target domain does help cross-domain recognition. As iter becomes larger, the mean accuracy rises and tends to be stable at certain point. An interesting phenomenon is that when iter gets too large, the performance would drop slightly. This result may be explained by the fact that we introduce the so-called high-confidence testing samples into training set; however, it could still be wrongly labeled. The proportion of samples that are predicted based on only well labeled samples is $\frac{1}{iter}$, then the proportion of samples that would be affected by noisy labels is $1-\frac{1}{iter}$. As iter gets bigger, noisy labels would affect more samples. A more comprehensive discussion about how the performance changes during testing is given in the following section.

## 5. Discussion

In this section, we aim to discuss the easy-to-hard testing deeply, and maybe, some points can be the direction for future research. We report the running times of the experiments.

### 5.1. Easy-To-Hard Testing vs. Single Testing

**Figure 6:**
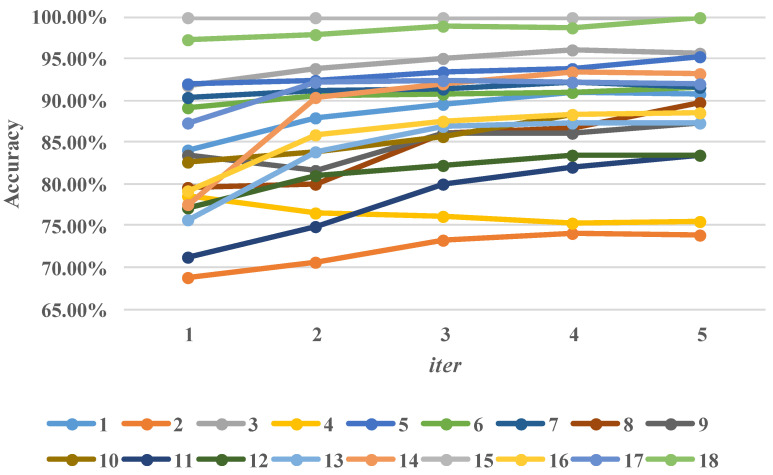
Performance (accuracy, %) of each subtask.

In the previous section, we showed that the mean accuracy increases as iter becomes larger, but whether the easy-to-hard testing is helpful for each subtask remains unclear. In Figure 6, we present how the accuracy changes for all the subtasks for each iteration; the number corresponds to the index in Table 3. Firstly, we define the easy task and the hard task by the initial accuracy. If the initial accuracy is high, the task is considered to be the easy task, and vice versa. We select 85% as the threshold roughly. Intuitively, we can say that the improvements for the hard task are more significant than for theeasy task, and the reason is that the difficulties in recognizing target samples in the hard task have greater differences, which is consistent with our hypothesis. Another interesting finding is that the easy-to-hard testing may have a negative influence on the hard tasks, e.g., Task No. 16 (W→C). This can be explained by the fact that the selected confident samples may still be wrongly labeled.

### 5.2. Computation Complexity and Running Time

The computational cost is detailed as follows: Considering Algorithm 1, $O(n_{s})$ is for source clustering using K-means and $O(n_{t})$ for testing. Use $N=n_{s}+n_{t}$ to denote the total number of source and target samples, then the computational complexity for single testing is O(N). When it comes to Algorithm 2, since it is a linear decreasing model, its computational complexity can be denoted as $O(\frac{1}{2}\cdot iter\cdot N)$. Since $\frac{1}{2}\cdot iter<<N$, the total computational complexity can be simplified to O(N), which means that the computational complexity increases linearly as the number of samples increases. For comparison, CAPLS and SPL have a complexity of $O(N^{3})$. When the number of samples becomes bigger, the proposed method would save much time.

We check the execution time of all the competing methods and report the total running time of 18 subtasks. All the methods were implemented with MATLAB 2017a and executed on the same computer, with I7-4790 CPU, 3.60 GHz, and 8 GB RAM. The results are shown in Table 4. Obviously, our method is the fastest algorithm except for the two baseline methods, NN and NCP. Besides, the proposed method is more than 1000 times faster than CAPLS and SPL with respect to the average running time, which is consistent with the previous analysis.

## 6. Conclusions and Future Works

In this paper, we propose a sample-guided adaptive class prototype method for visual domain adaptation. Unlike previous methods focusing on distribution matching, the proposed method aims to adjust the decision boundary according to the domain discrepancy existing in different samples. Extending the NCP, we present the modified NCP to explore a balance between single-sample discrimination and class-wise discrimination. For better exploiting the discriminative structures existing in the target domain, an east-to-hard training scheme is proposed. Target samples are considered to be of different difficulties to be recognized, then it selects the easy samples and uses them to make predictions for hard samples. Experimental results show that the proposed method is both effective and efficient. However, despite these promising results, questions remain. Previous works proved that class weight bias would gives rise to the performance degradation of statistic based methods, so our method (which does not rely on the statistics of the sample distribution) is expected to gain a large improvement on Office-Caltech10 than on ImageCLEF. However, the experimental results did not find a significant difference between these, and we think this can be explained by the changing sample size have more demand for an adaptive cluster number than the same sample size, while we adopted a fixed number of clusters for all the experiments.

Existing domain adaptation works study how to transfer knowledge between data from different sources; however, there is abundant room for further progress in transferring between different tasks. For example, if a person is good at poker, he/she may grasp chess quickly. We call that learning from experience, but currently, our models can only learn from data. Consequently, we believe investigating learning from experience is the key step to achieving artificial general intelligence.

## Figures and Tables

**Table 1:** Notations and descriptions used in this paper.

Notations	Description
$D_{s}/D_{t}$	source/target domain
$X_{s}/X_{t}\in R^{n_{s}/n_{t}\times m}$	source/target data
$Y_{s}/Y_{t}\in R^{n_{s}/n_{t}\times 1}$	source (available)/target (unavailable) labels
$n_{s}/n_{t}\in R$	# of source/target samples
m∈R	# of features
c∈R	# of classes
k∈R	# of clusters for each class
iter∈R	# of iterations for testing

**Table 2:** Image numbers in Office-Caltech.

Object	Backpack	Bike	Calculator	Headphones	Keyboard	Laptop	Monitor	Mouse	Mug	Projector
Caltech	151	110	100	138	85	128	133	94	87	97
Amazon	92	82	94	99	100	100	99	100	94	98
Webcam	29	21	31	27	27	30	43	30	27	30
DSLR	12	21	12	13	10	24	22	12	8	23

**Table 3:** Performance (accuracy %) on ImageCLEF (Cross Language Evaluation Forum (CLEF)) (Nos. 1–6) and Office-Caltech10 (Nos. 7–18). CAPLS, confidence-aware pseudo label selection; MASF, modified A-distance sparse filtering; GSMAX, generalized soft-max; SPL, selective pseudo labeling; DSF, discriminative sparse filtering; C, Caltech; I, ImageNet; P, Pascal; A, Amazon; W, webcam; D, DSLR.

No.	Task	NN	NCP	JDA	CAPLS	MASF	GSMAX	SPL	DSF	OURS
1	C→I	83.33	85.33	92.00	91.00	89.83	87.50	90.83	**93.16**	90.17 ± 0.55
2	C→P	70.05	70.73	75.50	77.33	72.83	70.39	**78.17**	75.63	74.04 ± 0.77
3	I→C	90.00	92.67	92.33	94.17	93.17	92.83	94.33	**95.67**	95.20 ± 0.32
4	I→P	75.47	76.99	77.00	75.80	76.83	**78.68**	77.50	77.49	76.99 ± 1.32
5	P→C	81.33	92.33	82.83	90.67	85.33	91.50	91.33	85.83	**93.77** ± 0.73
6	P→I	77.33	**90.67**	79.16	85.00	80.83	86.67	85.83	82.50	90.53 ± 1.02
7	C→A	85.70	91.23	89.77	**92.90**	90.81	92.48	92.80	91.12	92.05 ± 0.61
8	C→W	66.10	76.95	83.72	89.83	87.46	81.02	85.08	**91.52**	88.34 ± 1.14
9	C→D	74.52	82.17	86.62	91.08	89.81	89.81	**91.72**	89.17	89.68 ± 2.36
10	A→C	70.35	84.77	82.27	81.66	87.36	85.31	81.39	83.88	**88.16** ± 0.59
11	A→W	57.29	74.24	78.64	81.69	81.02	81.69	84.07	82.03	**84.95** ± 1.19
12	A→D	64.97	84.08	80.25	**90.45**	86.62	87.26	**90.45**	89.17	84.71 ± 2.43
13	D→C	60.37	72.31	83.52	**87.62**	85.04	81.39	74.00	81.92	86.16 ± 1.14
14	D→A	62.53	77.35	90.18	**92.38**	91.34	77.97	91.96	89.35	91.65 ± 2.07
15	D→W	98.73	95.54	**100.00**	**100.00**	99.36	97.45	**100.00**	**100.00**	99.49 ± 0.53
16	W→C	52.09	80.14	85.12	**89.05**	85.75	84.95	88.51	84.23	88.42 ± 0.38
17	W→A	62.73	86.01	91.44	**93.32**	90.40	90.61	**93.32**	91.44	92.34 ± 0.40
18	W→D	89.15	93.56	98.98	99.66	98.98	98.98	**100.00**	98.30	99.05 ± 1.06
19	AVG	73.45	83.73	86.07	89.09	87.38	86.47	88.41	87.91	**89.21**

**Table 4:** Running time (seconds) on ImageCLEF (Nos. 1–6) and Office-Caltech10 (Nos. 7–18).

No.	Task	NN	NCP	CAPLS	MASF	GSMAX	SPL	DSF	OURS
1	C→I	0.469	0.032	292.867	4.961	0.842	341.601	6.798	0.526
2	C→P	0.128	0.033	264.741	4.922	0.914	304.481	6.893	0.463
3	I→C	0.126	0.032	296.524	4.953	4.397	334.425	6.666	0.473
4	I→P	0.127	0.033	251.369	4.912	3.161	290.072	6.682	0.467
5	P→C	0.141	0.033	261.794	4.823	1.610	302.712	6.600	0.455
6	P→I	0.129	0.033	247.965	5.051	12.975	283.321	6.674	0.459
7	C→A	1.452	0.095	2516.933	90.814	3.936	3206.378	10.515	1.801
8	C→W	1.043	0.070	897.388	87.457	2.484	1030.175	6.755	1.282
9	C→D	0.966	0.061	842.526	89.808	2.677	921.195	5.905	1.210
10	A→C	1.297	0.095	3278.291	87.355	3.700	4365.437	11.097	1.774
11	A→W	0.806	0.060	2870.206	81.016	2.412	3528.711	5.879	1.070
12	A→D	0.786	0.054	2725.151	86.624	3.224	3272.032	4.716	0.998
13	D→C	0.703	0.062	735.878	85.040	2.229	833.277	6.479	0.850
14	D→A	0.562	0.052	1510.084	91.336	1.874	1840.393	5.528	0.770
15	D→W	0.290	0.022	319.998	99.363	1.612	369.574	1.951	0.347
16	W→C	0.633	0.055	519.365	85.752	1.764	622.318	5.375	0.716
17	W→A	0.509	0.046	1336.746	90.396	1.731	1751.001	4.678	0.620
18	W→D	0.221	0.022	241.107	98.983	1.481	278.883	1.982	0.322
19	SUM	10.388	0.890	19,408.930	1103.571	53.023	23,875.990	111.173	14.603
